# The impact of AI literacy on work–life balance and job satisfaction among university faculty: a self-determination theory perspective

**DOI:** 10.3389/fpsyg.2025.1669247

**Published:** 2025-09-17

**Authors:** Ling Huang, Yuping Zhao

**Affiliations:** ^1^School of Business and Tourism Management, Postdoctoral Research Station in Business Administration, Yunnan University, Kunming, China; ^2^General Education Center, Communication University of China, Beijing, China

**Keywords:** AI literacy, university faculty, self-determination theory, work–life balance, job satisfaction, technology acceptance

## Abstract

**Introduction:**

The emergence of artificial intelligence (AI) is transforming the nature of academic work, yet the role of AI literacy in supporting faculty well-being remains underexplored. This study investigates how AI literacy influences university faculty’s work-life balance and job satisfaction through the satisfaction of three basic psychological needs.

**Methods:**

Survey data were collected from 511 faculty members. Measures included AI literacy, perceived autonomy, perceived competence, perceived relatedness, work-life balance, job satisfaction, and technology acceptance. Statistical analyses examined the direct and indirect effects of AI literacy on faculty well-being.

**Results:**

The findings indicate that AI literacy significantly enhances the satisfaction of autonomy, competence, and relatedness. These, in turn, promote greater work-life balance. Further analysis shows that only perceived autonomy directly predicts job satisfaction, while competence and relatedness influence job satisfaction indirectly through work-life balance. Technology acceptance was found to moderate the relationship between AI literacy and psychological need fulfillment.

**Discussion:**

This study illuminates the psychological pathways through which AI literacy contributes to faculty well-being. It extends the application of Self-Determination Theory to technology-intensive academic settings and offers practical implications for designing AI literacy initiatives and faculty support strategies in higher education.

## 1 Introduction

The swift rise of artificial intelligence (AI) has reshaped how work is structured and performed in educational contexts (Zhang, 2023). In higher education, AI is increasingly embedded in curriculum design, instructional analytics, assessment feedback, and other core academic activities (Hwang et al., 2020). While these technologies enhance instructional efficiency, they also present new challenges, including evolving professional roles, continuous demands for upskilling, and increased risks of psychological strain and job burnout (Yu, 2024; Zhou J. S. et al., 2024). Faculty members must not only adapt to rapidly changing technologies but also manage elevated workloads and mounting psychological pressures. In this context, developing AI literacy—a composite of knowledge, attitudes, and competencies necessary to understand and apply AI tools—has become an essential skill set for academic professionals (Laupichler et al., 2022; Ng et al., 2023; Sperling et al., 2024).

Although existing research has begun to examine the relationship between AI literacy and educators’ professional experiences, key psychological dimensions remain insufficiently explored. For instance, Hashem et al. (2024) found that higher levels of AI literacy improve teaching effectiveness and reduce burnout, though their emphasis was primarily on technological performance. Similarly, Bhojak et al. (2025) reported a positive correlation between AI proficiency and job satisfaction, but did not investigate the underlying psychological mechanisms. Zheng and Zhang (2025) noted that the increased use of educational technology may blur work–family boundaries; however, their analysis lacked AI-specific focus and failed to address psychological needs explicitly.

Despite these preliminary insights, there remains a paucity of systematic empirical research on how AI literacy shapes university faculty’s psychological functioning, subjective well-being, and work–life balance (Ding et al., 2024). Both work–life balance and job satisfaction are crucial indicators of faculty well-being and are closely tied to mental and physical health, professional engagement, and long-term career sustainability (Landolfi et al., 2021).

Self-Determination Theory (SDT) provides a well-established framework for understanding these psychological processes. According to SDT, the satisfaction of three basic psychological needs—perceived autonomy, perceived competence, and perceived relatedness—is fundamental to intrinsic motivation, psychological well-being, and job satisfaction (Ryan and Deci, 2020). Prior studies suggest that satisfying these needs significantly influences individuals’ experiences with emerging technologies (Shen and Cui, 2024). Moreover, faculty members’ level of technology acceptance may moderate the extent to which AI literacy supports psychological need fulfillment (Pan, 2020).

Nonetheless, notable gaps persist. First, much of the literature focuses narrowly on performance-related outcomes, overlooking AI literacy’s potential role in meeting faculty members’ psychological needs (Bhojak et al., 2025; Xiao et al., 2025). Second, few studies have empirically validated the psychological mechanisms underpinning this relationship through the lens of SDT (Zhou J. S. et al., 2024; Zhou T. et al., 2024). Third, existing models often neglect the moderating influence of technology acceptance, limiting their explanatory power (Şimşek, 2025). Crucially, an integrative framework that brings together AI literacy, SDT, and technology acceptance is still lacking.

To address these gaps, the present study focuses on university faculty and investigates the following research questions: (1) Does AI literacy influence perceived autonomy, perceived competence, and perceived relatedness? (2) Do these psychological needs contribute to enhanced work–life balance and job satisfaction? (3) Does technology acceptance moderate these psychological pathways?

By integrating AI literacy with foundational psychological constructs, the study aims to identify key psychological constructs involved in faculty adaptation to the evolving demands of AI-mediated academic work. The findings offer theoretical insights and practical implications for promoting the sustainable and psychologically supportive adoption of AI technologies in higher education. This paper is organized into the following sections: theoretical background and literature review, research model and hypotheses, methodology, data analysis, and discussion.

## 2 Theoretical framework and literature review

### 2.1 AI literacy

To conceptualize artificial intelligence (AI) literacy in the context of higher education, a literature search and thematic analysis were conducted using the core search terms “AI literacy” and “teacher” in the Web of Science database. The results, summarized in Table 1, reveal that definitions of AI literacy are diverse and continuously evolving. Some scholars define AI literacy as the capacity to engage with AI tools effectively and ethically, critically assess their outputs, and adapt to rapidly changing technological environments (Kelley and Wenzel, 2025; Ozudogru and Durak, 2025). Others emphasize its interactive and collaborative dimensions, suggesting that AI literacy involves active engagement with AI systems that goes beyond mere technical proficiency or tool operation (Ayanwale et al., 2024).

**Table 1:** Summary of AI literacy conceptualizations and related research contributions.

Reference	Conceptual definition	Research method	Research contribution
Kelley and Wenzel (2025)	AI literacy refers to the capacity to engage with AI tools effectively and ethically, critically evaluate AI outputs, and flexibly adapt across diverse environments.	Qualitative Study	Conducted a systematic review of the multi-stage integration of generative AI in teacher education, distilling key practical experiences.
Ayanwale et al. (2024)	AI literacy is defined as a comprehensive ability to critically understand, appropriately apply, and actively engage with AI technologies across various contexts.	Quantitative Study	Examined the current status of AI literacy among pre-service teachers in Nigeria and proposed a localized teacher training framework, providing empirical evidence on technical and ethical dimensions.
Ning et al. (2025)	AI literacy encompasses the knowledge and application skills required for teachers to adapt to intelligent teaching environments, including perception, knowledge, skills, practical application, and ethics.	Quantitative Study	Developed and validated a measurement scale for teachers’ AI literacy, enriching the structural dimensions and assessment tools for AI literacy.
Sperling et al. (2024)	AI literacy refers to teachers’ integrated ability to combine AI knowledge, teaching skills, and ethical judgment in educational practice.	Qualitative Study	Conducted a systematic review of AI literacy in teacher education, identified research gaps, and proposed recommendations for in-situ AI literacy and ethical training.
Ozudogru and Durak (2025)	AI literacy is the ability to use AI products and tools and to assess their potential social and environmental impacts.	Quantitative Study	Developed and validated a structural path model linking pre-service teachers’ AI readiness to innovation, perceived threats, and AI literacy.
Al-Abdullatif (2024)	AI literacy integrates AI-related knowledge, skills, teaching, and assessment competencies.	Quantitative Study	Empirically investigated the key drivers of teachers’ adoption of generative AI, focusing on the relationships among Technology Acceptance, technological pedagogical content knowledge (TPACK) for intelligent technologies, AI literacy, and perceived trust.

Source(s): Created by author.

**Figure 1:**
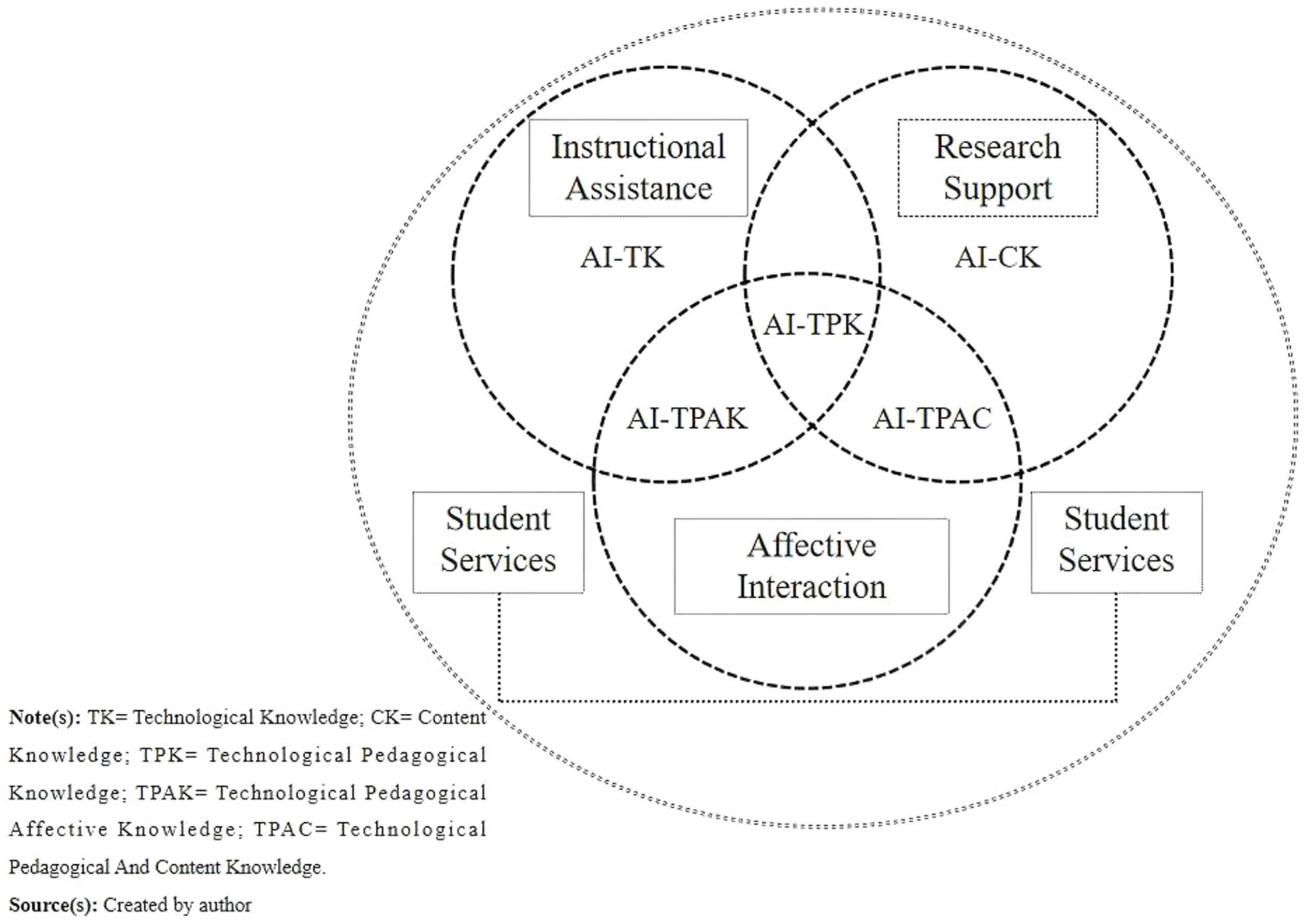
University faculty’s use of Al.

In higher education, faculty members serve as primary users of AI technologies in both teaching and research. Their AI literacy often manifests in complex, multidimensional ways (Laupichler et al., 2022). Drawing from prior literature and observed academic practices, faculty typically apply AI tools across four core domains: instructional assistance, research support, student services, and affective interaction (Yu, 2024; Zawacki-Richter et al., 2019). Their interactions with AI systems can be characterized through several common mechanisms (Şimşek, 2025; Kim, 2024), including: Information input–feedback, such as generating lesson content; Conflict resolution–trust building, for reconciling discrepancies between human and AI suggestions; Task delegation–cognitive offloading, involving the automation of repetitive or routine tasks; Decision support–human–machine collaboration, where AI contributes to complex academic analyses. These interaction patterns help operationalize how AI literacy manifests in faculty members’ daily practices. As illustrated in Figure 1, AI literacy is distributed across intersecting domains of technological (TK), content (CK), and pedagogical (PK) knowledge, giving rise to integrated competencies such as AI-TPK, AI-TPAC, and AI-TPAK. This framework highlights the dynamic and interdisciplinary nature of AI literacy in higher education (see Figure 1).

An expanding body of research identifies AI literacy as a critical competency for instructional design, pedagogical decision-making, and student-centered learning. It plays an essential role in curriculum development, formative assessment, and personalized instruction (Al-Abdullatif, 2024; Ning et al., 2025). Several scholars further advocate for a broadened conceptualization of teacher AI literacy that integrates technical knowledge, pedagogical strategies, and ethical reasoning—thus emphasizing interdisciplinary thinking and reflective practice (Sperling et al., 2024). While these contributions have significantly advanced the conceptual landscape of AI literacy in education, much of the existing literature remains focused on operational skills. Relatively few studies explore the psychological or occupational dimensions of AI literacy, such as its impact on educators’ motivation, emotional states, or overall professional experience.

Building on the reviewed literature, the present study adopts a comprehensive definition proposed by Laupichler et al. (2022), Ng et al. (2023), and Sperling et al. (2024). University faculty’s AI literacy is defined as the systematic understanding and practical application of AI principles, tools, ethical considerations, and implementation strategies. Its core dimensions include technical proficiency, algorithmic thinking, interdisciplinary integration, sensitivity to educational equity, and awareness of AI’s broader societal implications (Abulibdeh et al., 2024). Faculty members with higher AI literacy typically demonstrate more favorable attitudes toward technology and greater competence in integrating AI into their professional roles, which in turn enhances teaching quality and research productivity (Bewersdorff et al., 2025). Therefore, AI literacy should not be conceptualized solely as a technical skill set, but rather as a psychologically meaningful framework that captures university faculty’s cognitive, emotional, and professional engagement with emerging technologies.

### 2.2 Self-determination theory

Self-Determination Theory (SDT), initially proposed by Ryan and Deci (2020), offers a foundational lens for examining human motivation and psychological well-being. At the core of SDT is the assertion that individuals possess three innate psychological needs—perceived autonomy, perceived competence, and perceived relatedness—whose fulfillment is essential for fostering intrinsic motivation, optimal functioning, and mental health (Janssen et al., 2013). Perceived autonomy involves volitional behavior and self-guided action; perceived competence reflects individuals’ beliefs in their abilities to successfully perform tasks; and perceived relatedness pertains to the sense of meaningful connection and support from others (Deci and Ryan, 2000).

These needs have been consistently associated with key professional outcomes in educational settings, including job satisfaction, psychological well-being, and organizational commitment (Inigo and Raufaste, 2019). In higher education, faculty autonomy in selecting and utilizing digital tools has been shown to enhance intrinsic motivation (Zheng et al., 2025). Likewise, confidence in using educational technology can reduce anxiety and improve engagement in teaching activities (Klassen and Chiu, 2010), while the satisfaction of relatedness needs fosters emotional support and strengthens faculty members’ sense of belonging within academic communities (Naidoo and Wagner, 2020).

Recent research has increasingly applied SDT to technology adoption contexts, particularly in exploring how faculty adapt to AI-integrated teaching and learning environments (Francis et al., 2024). These studies suggest that the degree to which AI innovations support or undermine psychological need satisfaction is essential in influencing educators’ attitudes, behaviors, and well-being.

Taken together, SDT provides a comprehensive and empirically grounded lens for examining the psychological mechanisms that underlie faculty engagement with AI technologies. It also offers a compelling theoretical foundation for understanding how AI literacy—as both a cognitive and behavioral construct—can influence motivation, job satisfaction, and broader professional experiences.

### 2.3 Work–life balance

Work–life balance refers to an individual’s ability to manage and reconcile the competing demands of professional and personal life in a satisfying and sustainable manner (Greenhaus and Beutell, 1985). University faculty often face significant role strain and time pressure due to their multiple responsibilities in teaching, research, and service (Pasamar et al., 2020). Effective time management and allocation of cognitive and emotional resources are thus critical to their psychological well-being (Pace et al., 2021).

AI technologies offer promising tools to alleviate academic workload and enhance efficiency, potentially affording faculty greater flexibility and control over their time (Bhojak et al., 2025). For example, automated grading systems and intelligent scheduling platforms can streamline repetitive tasks and improve task allocation, thereby helping faculty manage the boundary between work and personal life more effectively (Badri, 2024). However, in the absence of adequate institutional support, technological integration may give rise to new stressors—such as cognitive overload, digital fatigue, and increased anxiety—which may undermine rather than enhance work–life balance (Dorenkamp and Ruhle, 2019).

### 2.4 Job satisfaction

Job satisfaction is a multidimensional construct that reflects an individual’s overall appraisal of their work experience, encompassing aspects such as task content, work environment, interpersonal relationships, and career development opportunities. It is widely recognized as a key indicator of professional well-being and engagement among faculty (Troesch and Bauer, 2017). Both extrinsic factors (e.g., salary, institutional policies) and intrinsic factors (e.g., teaching motivation, academic identity) influence job satisfaction levels (Layek and Koodamara, 2024).

In the context of increasing digitalization in higher education, AI literacy has emerged as an important predictor of job satisfaction (Jose et al., 2025). Faculty members with higher levels of AI literacy often report a greater sense of control, efficacy, and competence in navigating digital teaching environments, which in turn enhances engagement and fulfillment (Ji et al., 2025). Moreover, AI tools that automate routine tasks and streamline workflows can also boost productivity and reinforce a sense of accomplishment (Xia et al., 2022). However, disparities in technological readiness can lead to anxiety, information overload, and emotional exhaustion—factors that detract from overall job satisfaction (Li and Yu, 2022).

### 2.5 Technology acceptance

Technology acceptance is commonly defined as an individual’s evaluation of and readiness to embrace new technologies, often framed by two key constructs: perceived usefulness and ease of use (Venkatesh and Davis, 2000). In educational settings, technology acceptance plays a pivotal role in shaping faculty members’ adoption behavior and their emotional responses to digital tools (Scherer et al., 2019).

AI technologies, while potentially transformative, often present barriers to acceptance due to their complexity and ethical concerns related to privacy, transparency, and accountability (Bergdahl et al., 2023). Conversely, educators with high levels of technology acceptance are more likely to engage in active learning, integrate innovative tools into their teaching, and demonstrate greater openness to pedagogical experimentation (Racero et al., 2020).

Importantly, technology acceptance may also moderate the relationship between AI literacy and psychological outcomes. It can shape how faculty experience autonomy, competence, and emotional responses when implementing AI in their instructional and research practices (Antonietti et al., 2022). Understanding the role of technology acceptance is therefore essential for designing effective faculty development programs and for promoting sustainable and psychologically supportive AI integration in higher education.

## 3 Research model and hypothesis development

### 3.1 The impact of AI literacy on self-determination theory constructs: perceived autonomy, perceived competence, and perceived relatedness

AI literacy, beyond operational proficiency, represents faculty members’ ability to self-direct technology use, critically evaluate AI-generated outputs, and integrate tools into pedagogical practices (Celik, 2023). High AI literacy equips teachers to select suitable AI functions, customize workflows, and adapt teaching strategies without relying heavily on external guidance (Chiu and Chai, 2020). This capability fosters perceived autonomy because decisions about technology use are internally regulated rather than externally imposed (Ryan and Deci, 2000). Autonomy emerges when individuals experience volition in aligning AI applications with their instructional goals, reducing feelings of technological constraint. Conversely, low AI literacy can lead to dependency on preset tools or institutional mandates, limiting choice and control. The presence of high AI literacy therefore strengthens the sense of ownership over instructional processes, enabling faculty to exercise freedom in technology adoption and thereby enhancing self-determined engagement in AI-enhanced education (Zawacki-Richter et al., 2019). Accordingly, we propose:

*H1*: AI literacy has a significant positive effect on university faculty’s perceived autonomy in using AI.

Perceived competence reflects the belief in one’s ability to effectively perform tasks (Ryan and Deci, 2000). In AI-supported higher education, this belief is strengthened when teachers possess the necessary technical and cognitive skills to translate AI capabilities into academic outcomes (Xia et al., 2023). Faculty with high AI literacy can manage data-driven analytics, apply intelligent feedback, and integrate cross-platform resources efficiently. These abilities reduce uncertainty when facing complex tasks, reinforcing task mastery and professional efficacy (Wang et al., 2025). Mastery experiences with AI tools also create positive performance feedback loops, increasing confidence and willingness to undertake more challenging projects. In contrast, insufficient AI literacy may result in trial-and-error inefficiency, eroding competence perceptions. Thus, AI literacy operates as a foundational resource that transforms technical knowledge into tangible achievements, directly enhancing teachers’ confidence in their technological and pedagogical capabilities. Therefore, we propose:

*H2*: AI literacy has a significant positive effect on university faculty’s perceived competence in using AI.

Perceived relatedness, as defined in Self-Determination Theory, reflects the need to feel connected to others and experience mutual support (Ryan and Deci, 2000). AI literacy enhances relatedness by enabling faculty to participate effectively in technology-mediated collaboration, such as co-teaching, shared resource creation, and engagement in virtual scholarly communities (Ng et al., 2023). Proficiency in AI tools facilitates smooth communication, efficient content sharing, and mutual problem-solving, which strengthen interpersonal trust and social bonds (Singh and Aziz, 2025). When teachers can competently navigate AI-enhanced platforms, they are more likely to contribute meaningfully to joint projects, receive peer recognition, and build sustained professional relationships. This socially embedded use of AI fosters organizational belonging and reinforces collective identity (Wang et al., 2025; Xia et al., 2022). Conversely, low AI literacy can hinder participation in collaborative environments, reducing opportunities for connection and support. Accordingly, we propose:

*H3*: AI literacy has a significant positive effect on university faculty’s perceived relatedness in using AI.

### 3.2 The impact of perceived autonomy on work–life balance and job satisfaction

Perceived autonomy reflects the extent to which individuals feel free to make choices and regulate their actions according to personal goals (Ryan and Deci, 2000). In academic contexts, this sense of volition enables faculty to organize tasks, prioritize responsibilities, and adjust teaching schedules in ways that align with personal circumstances (Van den Broeck et al., 2016). Within AI-assisted environments, autonomy manifests when teachers can independently determine how and when to integrate AI tools, select functionalities suited to their needs, and adapt outputs for specific pedagogical purposes (Wu et al., 2024). Such flexibility reduces time pressure, minimizes role conflict, and enhances the ability to allocate resources between work and personal life (Khawand and Zargar, 2022). Greater autonomy also supports proactive coping strategies, allowing educators to manage workload without compromising personal well-being (Fotiadis et al., 2019). These mechanisms explain why autonomy in AI use is likely to facilitate a more sustainable work–life balance for faculty members. Therefore, we propose:

*H4*: University faculty’s perceived autonomy in using AI has a significant positive effect on their work–life balance.

Autonomy in AI-supported teaching fosters a sense of control over instructional decisions, enabling faculty to select content, pedagogical strategies, and technological configurations that best serve their objectives (Gagné et al., 2022). This self-directed approach strengthens goal alignment, reinforces intrinsic motivation, and enhances professional purpose (Ma and Vu, 2024). In personalized teaching contexts, AI tools allow real-time adaptation of learning materials, automated assessment, and targeted feedback, enabling teachers to implement innovations without excessive external constraints (Cho and Jung, 2025). Positive experiences with such autonomy can increase satisfaction through improved student outcomes and professional recognition. By contrast, limited control over AI integration may generate frustration or reduce engagement. The ability to decide when and how to use AI therefore directly contributes to job satisfaction by reinforcing educators’ sense of competence, self-worth, and alignment with institutional goals. Accordingly, we propose:

*H5*: University faculty’s perceived autonomy in using AI has a significant positive effect on their job satisfaction.

### 3.3 The impact of perceived competence on work–life balance and job satisfaction

Perceived competence describes individuals’ confidence in their ability to meet situational demands effectively (Deci and Ryan, 2000). In AI-enhanced teaching environments, competent faculty can quickly identify appropriate tools, adapt them to diverse instructional needs, and transfer learned skills across tasks (Celik, 2023). This efficiency reduces trial-and-error inefficiencies, allowing more time for strategic planning and personal activities. Competence also supports better workload structuring, helping faculty manage the boundary between work and non-work domains (Yin and Huang, 2021). Automation capabilities, such as grading algorithms and scheduling assistants, further improve resource allocation and reduce repetitive tasks (Ayanwale et al., 2024). As educators gain mastery over AI, they are more likely to experience control over their professional routines, freeing time for personal commitments and improving overall life balance. Therefore, we propose:

*H6*: University faculty’s perceived competence in using AI has a significant positive effect on their work–life balance.

Competence contributes to higher self-efficacy, enabling educators to set challenging goals and maintain confidence in achieving them (Klassen and Chiu, 2010). In AI-supported contexts, skill growth can lead to improved teaching quality, innovative research outputs, and enhanced operational control (Wang et al., 2025). Mastery experiences with AI reduce apprehension toward technological change, facilitating adaptability and reducing work-related stress (Hofer et al., 2021). Competent educators are also more likely to receive positive feedback from peers, students, and institutions, reinforcing a sense of accomplishment. Personalized AI applications, such as adaptive learning platforms, can further increase recognition by highlighting the impact of teachers’ expertise on student performance (Molefi et al., 2024). These factors collectively strengthen job satisfaction by fulfilling psychological needs for achievement and professional growth. Accordingly, we propose:

*H7*: University faculty’s perceived competence in using AI has a significant positive effect on their Job Satisfaction.

### 3.4 The impact of perceived relatedness on work–life balance and job satisfaction

Within Self-Determination Theory, relatedness reflects the need to feel connected and supported by others (Deci and Ryan, 2000). In AI-integrated higher education, strong relatedness allows faculty to form cooperative networks that buffer the uncertainty and stress of adopting new technologies (Prenger et al., 2017). Engagement in collaborative teaching, co-development of AI-supported learning resources, and participation in online academic communities facilitates the exchange of both technical and emotional resources (Ng et al., 2023). Such networks reduce the cognitive load of technology use, enhance mutual trust, and create a supportive environment for managing workload. Social bonds also aid emotional regulation and promote adaptive coping, which preserves energy for non-work activities (Ertiö et al., 2024). Through these mechanisms, perceived relatedness enables faculty to sustain an equilibrium between professional and personal life, thereby enhancing work–life balance. Therefore, we propose:

*H8*: University faculty’s perceived relatedness in using AI has a significant positive effect on their work–life balance.

Supportive professional relationships contribute to a sense of belonging and recognition, which strengthens academic identity and motivation (Klassen et al., 2012). In AI-supported environments, shared platforms enable faculty to collaborate on innovative teaching designs, exchange strategies for AI integration, and celebrate collective achievements (Dilek et al., 2025). These interactions reinforce shared goals, reduce isolation, and increase emotional engagement in academic work (Wang et al., 2025). A strong sense of relatedness also provides a stable psychological foundation for navigating challenges in AI adoption, as mutual support reduces stress and enhances confidence. Furthermore, expanded academic networks foster opportunities for interdisciplinary collaboration and professional growth, which in turn heightens organizational belonging (Singh and Aziz, 2025). By reinforcing both emotional satisfaction and professional accomplishment, perceived relatedness serves as a psychological driver of job satisfaction in AI-integrated teaching and research contexts. Therefore, we propose:

*H9*: University faculty’s perceived relatedness in using AI has a significant positive effect on their job satisfaction.

### 3.5 The impact of work–life balance on job satisfaction

Work–life balance is essential for sustaining professional well-being because it mitigates role conflict and reduces burnout (Prasad and Pasupathi, 2025). Faculty who manage work and personal responsibilities effectively can maintain emotional stability and preserve cognitive resources for teaching and research (Cao et al., 2020). Balanced schedules promote a sense of control, enabling educators to respond more constructively to academic challenges. In AI-integrated environments, automation tools streamline grading, scheduling, and data analysis, thereby reducing time spent on repetitive tasks and freeing capacity for personal and family activities (Meharunisa et al., 2024). This efficiency creates a reinforcing cycle in which improved balance enhances overall satisfaction with work, and higher satisfaction further motivates effective time management. Faculty who sustain this equilibrium often report greater role clarity, stronger engagement, and a deeper sense of professional accomplishment (Wei and Ye, 2022). Therefore, we propose:

*H10*: University faculty’s job satisfaction is significantly and positively influenced by their work–life balance.

### 3.6 The moderating role of technology acceptance

Technology acceptance, as conceptualized in the Technology Acceptance Model, captures individuals’ perceptions of ease of use, perceived usefulness, and readiness to engage with new tools (Venkatesh et al., 2003). In the context of AI-enhanced higher education, high acceptance enables faculty to translate AI literacy into meaningful, autonomous use of technology. When teachers perceive AI as valuable and manageable, they are more likely to experiment with functions, adapt tools to personal teaching styles, and initiate independent problem-solving (Chiu et al., 2024). This strengthens their perceived control over technological processes and their freedom to design instructional approaches. Conversely, low acceptance can lead to avoidance behaviors, heightened anxiety, and underutilization of existing AI skills (Teo, 2011). In such cases, AI literacy may remain a latent capability rather than an active driver of autonomy. Technology acceptance thus shapes the psychological translation of AI knowledge into self-determined teaching behaviors (Dahri et al., 2024). Therefore, we propose:

*H11*: Technology acceptance moderates the positive relationship between AI literacy and perceived autonomy in using AI.

Faculty with high technology acceptance are more inclined to devote time and cognitive effort to mastering AI systems, which allows them to achieve operational proficiency and receive timely performance feedback (Scherer et al., 2019). This active engagement fosters a deeper understanding of AI functionalities and facilitates effective application in both teaching and research. When acceptance is high, AI literacy is more efficiently converted into perceived competence, reinforcing professional self-efficacy and enabling teachers to tackle complex academic tasks with confidence (Wang et al., 2025). In contrast, low acceptance often leads to neglect of learning opportunities, reluctance to apply existing AI knowledge, and limited skill growth (Sumak et al., 2011). Even when technical capabilities exist, resistance toward technology may hinder the translation of knowledge into effective practice. Thus, technology acceptance determines the extent to which AI literacy can be transformed into a tangible sense of competence in professional contexts. Therefore, we propose:

*H12*: Technology acceptance moderates the positive relationship between AI literacy and perceived competence in using AI.

Perceived relatedness in AI integration depends not only on technical knowledge but also on willingness to engage in technology-mediated collaboration (Ng et al., 2023). Faculty with high technology acceptance tend to view AI platforms as effective tools for interaction, resource sharing, and collective problem-solving (Kaliisa et al., 2022). This positive orientation encourages participation in interdisciplinary networks, joint curriculum design, and virtual academic communities, which strengthens interpersonal bonds and mutual trust. In contrast, low acceptance can result in avoidance of AI-based interactions, reduced collaborative initiatives, and diminished exposure to diverse perspectives (Teo and Noyes, 2014). Over time, such withdrawal limits opportunities for social support and weakens organizational connectedness. By shaping the frequency and quality of AI-mediated exchanges, technology acceptance influences how AI literacy contributes to the satisfaction of relatedness needs and the development of a sense of belonging in academic settings. Therefore, we propose:

*H13*: Technology acceptance moderates the positive relationship between AI literacy and perceived relatedness in using AI.

**Figure 2:**
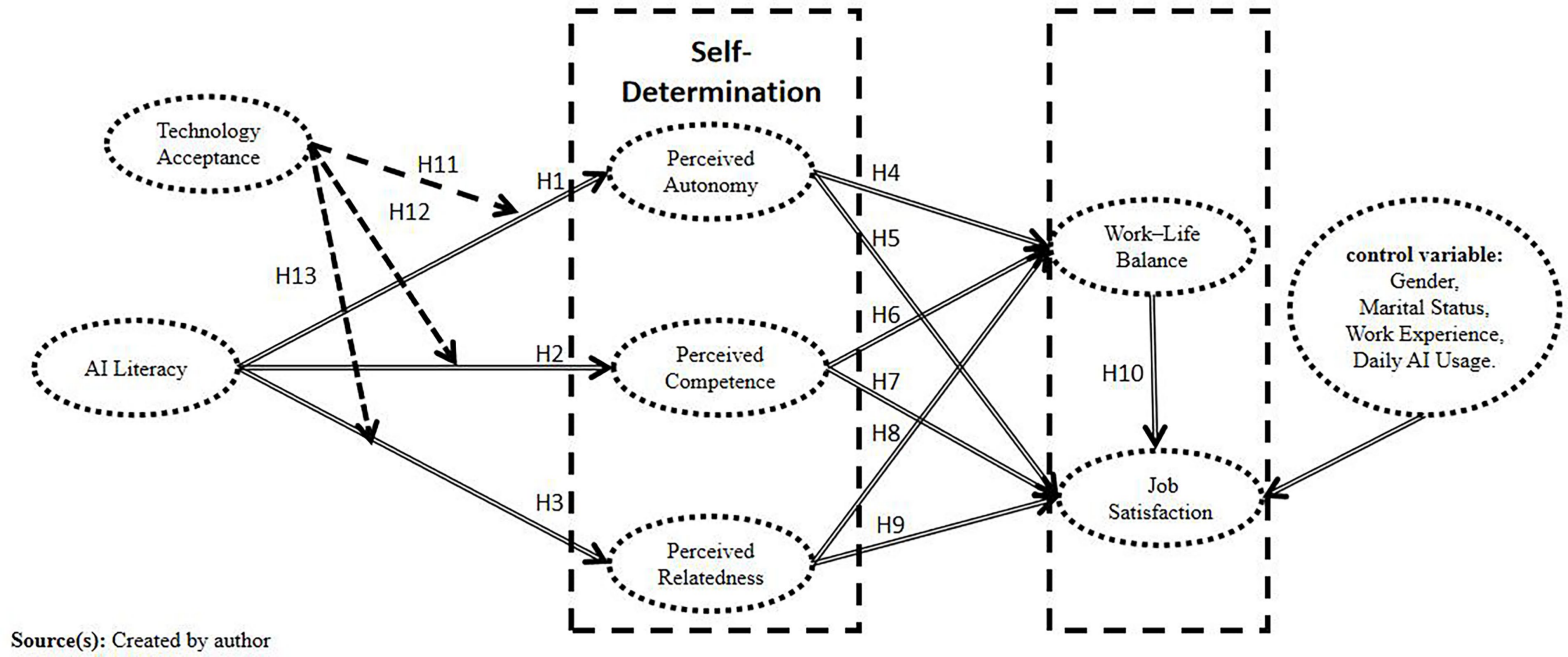
Research model.

These hypothesized relationships are synthesized in the research model depicted in Figure 2.

## 4 Method

### 4.1 Participants

Data for this study were collected via an online questionnaire administered through Wenjuanxing^1^, a commonly used platform in China. The instrument employed a seven-point Likert scale and was distributed using a stratified random sampling strategy. Participants were recruited through the platform’s representative sampling service, ensuring diverse and demographically balanced responses. All surveys were completed electronically. To incentivize participation, respondents who submitted valid questionnaires received a monetary reward of 5 RMB. To ensure data quality and participant relevance, the study implemented multiple inclusion and exclusion criteria. (1) Screening Questions: At the beginning of the questionnaire, two mandatory screening items were included to confirm eligibility: (a) “What is your current occupation?”—only respondents selecting “university faculty member” were included; and (b) “Do you have basic experience using AI tools?”—only those responding “Yes” proceeded to the main section. Failure to meet either criterion led to immediate exclusion. (2) Attention Check: A directed-response item was embedded mid-questionnaire (e.g., “To confirm you are paying attention, please select ‘Strongly disagree’ for this item.”). Participants who failed to respond as instructed were excluded from the final dataset. (3) Responses exhibiting identical selections across all Likert-scale items were flagged for satisficing behavior or inattentive responding and subsequently removed from the analysis. In total, 543 questionnaires were received. After removing 32 invalid responses based on the criteria above, 511 valid responses remained, resulting in an effective response rate of 94.11%. A summary of participant demographic characteristics is presented in Table 2.

**Table 2:** Demographic characteristics of participants.

Variable	Category	Frequency	Percentage
Gender	Male	209	40.90%
	Female	302	59.10%
Marital status	Married	288	56.36%
	Unmarried	223	43.64%
Work experience	0–5 years	110	21.53%
	6–10 years	198	38.75%
	11–15 years	130	25.44%
	16–20 years	47	9.20%
	More than 21 years	26	5.09%
Daily AI usage	0–1 h	178	34.83%
	1–3 h	143	27.98%
	3–5 h	115	22.50%
	5–7 h	53	10.37%
	More than 7 h	22	4.31%

Source(s): Created by author.

### 4.2 Measures

Measurement instruments in this research were derived from established scales with prior empirical validation across domestic and international studies (Haw et al., 2024; Lin et al., 2025; Mulyani et al., 2021; Wang et al., 2023; Yildirim et al., 2024). All scales were appropriately modified to align with the specific context of university faculty. The core variables measured in this study included AI literacy (AIL), perceived autonomy (PA), perceived competence (PC), perceived relatedness (PR), work–life balance (WLB), job satisfaction (JS), and technology acceptance (TA). Detailed information regarding the measurement dimensions, sample items, and sources of each scale is provided in Appendix 1.

### 4.3 Common method bias assessment

Given that all variables in this study were measured through cross-sectional self-reported questionnaires, procedural remedies were implemented to reduce the likelihood of common method variance (CMV). These included ensuring respondent anonymity, counterbalancing the order of measurement items, and embedding an attention-check question to minimize socially desirable responding and inattentive answering. To statistically assess CMV, Harman’s single-factor test was performed by entering all measurement items into an unrotated exploratory factor analysis. Results showed that the first factor explained 38.844% of the total variance, which is below the commonly accepted threshold of 40%, suggesting that CMV was not a severe issue (Podsakoff et al., 2003). In addition, a common latent factor approach was employed in SmartPLS to detect potential method effects. The variance inflation factors (VIFs) for all items were below 3.3, indicating that multicollinearity was not a concern and CMV was unlikely to bias the study’s results.

## 5 Data analysis and results

This study constructed a structural equation model (SEM) to examine the pathways through which AI literacy influences work–life balance and job satisfaction among university faculty. Descriptive statistics and preliminary analyses were conducted using SPSS 27.0, followed by SEM and path analysis using SmartPLS 4.0. In SEM methodology, covariance-based SEM (CB-SEM) is commonly used for confirmatory theory testing and evaluating global model fit, whereas partial least squares SEM (PLS-SEM) focuses on maximizing predictive accuracy and is especially appropriate for complex models, non-normal data, and exploratory research contexts. In this study, PLS-SEM was selected for four primary reasons: (1) the model includes seven latent constructs, multiple indicators, and a moderating effect, resulting in high structural complexity; (2) preliminary diagnostics revealed slight deviations from multivariate normality; (3) the research adopts an exploratory path-testing orientation aimed at extending rather than strictly confirming existing theory; and (4) PLS-SEM offers robustness and predictive power particularly suited to emerging research domains such as AI literacy in higher education. This choice is consistent with established methodological guidelines (Dash and Paul, 2021), which recommend PLS-SEM in early-stage theoretical model development and when prediction is a key goal.

### 5.1 Measurement model assessment

Validity was examined through three dimensions: content, convergent, and discriminant validity. Content validity was ensured by adapting measurement items from extensively validated scales in prior research. Convergent validity was supported by factor loadings (≥0.70) (Raykov and Marcoulides, 2019) and Average Variance Extracted (AVE ≥ 0.50), in line with Cheung et al. (2024). Discriminant validity was evaluated using the Heterotrait–Monotrait ratio (HTMT), with the recommended threshold set below 0.85.

As shown in Tables 3, 4, the measurement model demonstrated satisfactory reliability and validity. All latent variables exhibited Cronbach’s *α* values ranging from 0.903 to 0.947 and CR values ranging from 0.928 to 0.955, indicating strong internal consistency. Factor loadings fell within the range of 0.777 to 0.879, while AVE values ranged between 0.664 and 0.732, satisfying the criteria for convergent validity. HTMT values for all variable pairs were below 0.85, indicating good discriminant validity among the constructs.

**Table 3:** Results of reliability and convergent validity testing.

Latent variable	Measurement items	Mean	Standard deviation	Factor loading	Cronbach’s α	CR	AVE
AIL	AIL1	4.885	1.530	0.837	0.947	0.955	0.704
	AIL2	4.867	1.573	0.848			
	AIL3	4.793	1.583	0.840			
	AIL4	4.824	1.509	0.864			
	AIL5	4.765	1.548	0.806			
	AIL6	4.863	1.486	0.841			
	AIL7	4.691	1.529	0.826			
	AIL8	4.806	1.597	0.858			
	AIL9	4.828	1.510	0.829			
PA	PA1	4.941	1.544	0.872	0.906	0.930	0.726
	PA2	4.886	1.613	0.879			
	PA3	4.810	1.584	0.848			
	PA4	4.869	1.483	0.848			
	PA5	4.773	1.499	0.813			
PC	PC1	4.806	1.483	0.855	0.908	0.932	0.732
	PC2	4.750	1.458	0.861			
	PC3	4.943	1.567	0.847			
	PC4	4.932	1.595	0.858			
	PC5	4.693	1.502	0.856			
PR	PR1	4.722	1.474	0.856	0.903	0.928	0.719
	PR2	4.624	1.501	0.850			
	PR3	4.708	1.432	0.831			
	PR4	4.722	1.473	0.856			
	PR5	4.841	1.507	0.848			
WLB	WLB1	4.730	1.569	0.841	0.941	0.951	0.709
	WLB2	4.793	1.488	0.832			
	WLB3	4.605	1.545	0.849			
	WLB4	4.712	1.523	0.855			
	WLB5	4.683	1.544	0.856			
	WLB6	4.769	1.533	0.855			
	WLB7	4.765	1.525	0.841			
	WLB8	4.724	1.505	0.804			
JS	JS1	5.121	1.392	0.839	0.903	0.928	0.721
	JS2	5.155	1.435	0.870			
	JS3	5.182	1.389	0.850			
	JS4	5.147	1.382	0.850			
	JS5	5.213	1.464	0.838			
TA	TA1	4.781	1.480	0.830	0.937	0.947	0.664
	TA2	4.777	1.494	0.806			
	TA3	4.886	1.397	0.833			
	TA4	4.810	1.464	0.837			
	TA5	4.863	1.512	0.840			
	TA6	4.977	1.389	0.818			
	TA7	4.853	1.480	0.809			
	TA8	4.820	1.447	0.781			
	TA9	4.814	1.443	0.777			

Source(s): Created by author.

**Table 4:** Results of discriminant validity testing.

Variable	AIL	PA	PC	PR	WLB	JS	TA
AIL							
PA	0.542						
PC	0.588	0.627					
PR	0.565	0.573	0.608				
WLB	0.564	0.600	0.600	0.580			
JS	0.389	0.475	0.410	0.373	0.480		
TA	0.406	0.430	0.393	0.403	0.446	0.627	

Source(s): Created by author.

### 5.2 Structural model analysis

Before testing the hypothesized structural relationships, four control variables—gender, marital status, work experience, and daily AI usage—were included in the model to account for potential confounding effects on job satisfaction. The results showed that none of these control variables had a statistically significant impact on job satisfaction (all *p* > 0.05), indicating that the subsequent path estimates are unlikely to be biased by these demographic or usage-related factors.

#### 5.2.1 Model explanatory power and predictive relevance

Table 5 presents the coefficients of determination (*R*^2^) and predictive relevance (*Q*^2^) for the endogenous variables. The *R*^2^ values for perceived autonomy (0.315), perceived competence (0.337), perceived relatedness (0.345), work–life balance (0.435), and job satisfaction (0.253) all reached acceptable levels, with the explanatory power for work–life balance being particularly strong. All *Q*^2^ values were greater than zero (ranging from 0.218 to 0.342), indicating that the model demonstrates satisfactory predictive relevance. These results support the robustness and theoretical validity of the model.

**Table 5:** Explanatory power (*R*^2^) and predictive relevance (*Q*^2^) of the structural model.

Endogenous Latent Variables	*R* ^2^	*Q* ^2^
PA	0.315	0.303
PC	0.337	0.323
PR	0.345	0.327
WLB	0.435	0.342
JS	0.253	0.218

Source(s): Created by author.

#### 5.2.2 Path coefficient analysis and hypothesis testing

**Figure 3:**
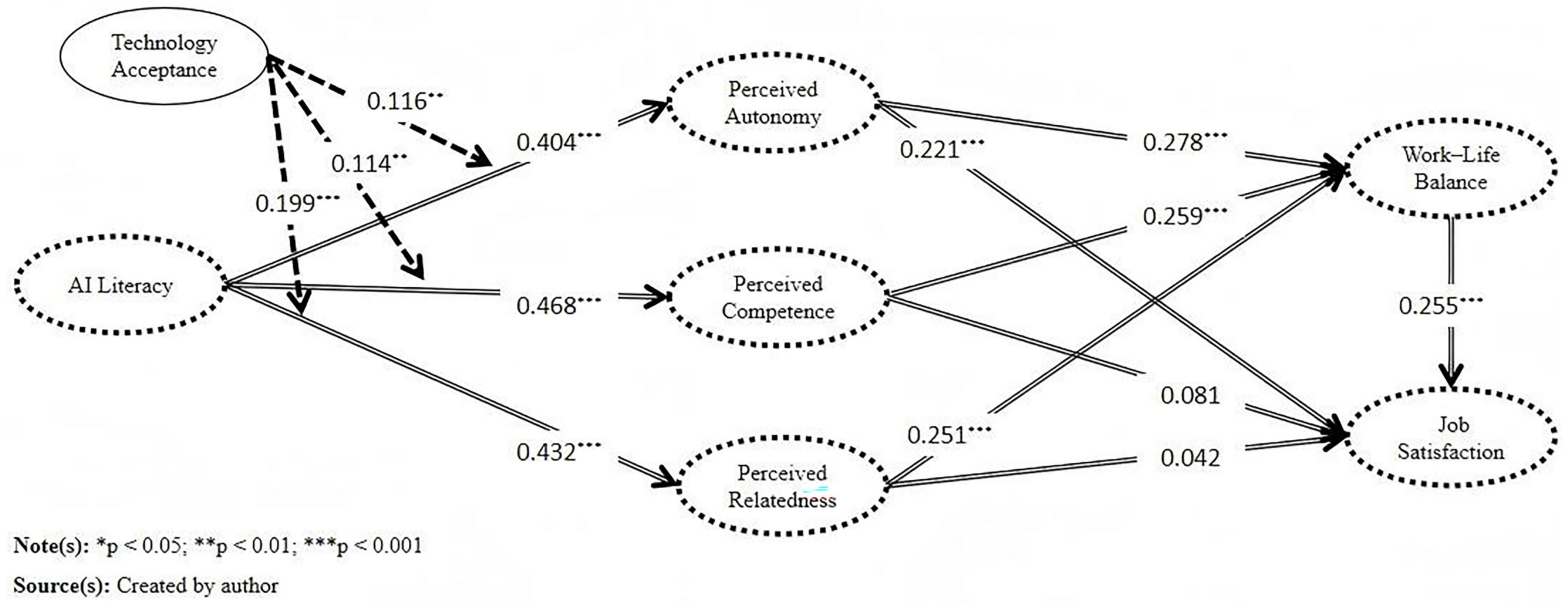
Path analysis results of the structural equation model.

Figure 3 illustrates the structural model outcomes, indicating that AI literacy significantly and positively influenced perceived autonomy (β=0.404,p<0.001), perceived competence (β=0.468,p<0.001), and perceived relatedness (β=0.432,p<0.001), providing strong support for H1, H2, and H3. These results suggest that higher levels of AI literacy among university faculty are associated with enhanced experiences of self-control, competence fulfillment, and social connectedness.

Further analysis revealed that Perceived Autonomy (β=0.278,p<0.001), Competence (β=0.259,p<0.001), and Relatedness (β=0.251,p<0.001) all significantly and positively influenced work–life balance, supporting H4, H6, and H8. These findings indicate that when faculty members’ psychological needs are met, they are more capable of balancing teaching, research, and personal life, which helps reduce role conflicts. Regarding the impact on job satisfaction, only perceived autonomy showed a significant positive effect (β=0.221,p<0.001), supporting H5. The effects of perceived competence (β=0.081,p=0.186) and perceived relatedness (β=0.042,p=0.471) were not statistically significant, and thus H7 and H9 were not supported. Several potential explanations for these results have been suggested in the literature. Li et al. (2025) noted that university faculty’s job satisfaction is strongly influenced by organizational factors such as performance evaluations, work environment, promotion pathways, and career support, which may dilute the direct impact of perceived competence. Chang et al. (2024) emphasized that the pressures brought by technological penetration may counteract its positive effects. Lyu and Zhu (2019), as well as Yang and Ling (2023), argued that university teaching tends to be relatively independent, with lower frequencies of social interaction, making it difficult for perceived relatedness to have a significant effect on satisfaction. Kim (2024) further pointed out that current AI tools in education primarily focus on enhancing individual efficiency, while their capacity to support social interaction and collaborative work remains underdeveloped.

Additionally, work–life balance was found to be a significant positive predictor of job satisfaction (β=0.255,p<0.001), providing support for H10. This suggests that faculty members’ positive evaluations of their work are closely tied to their ability to effectively integrate work and life roles.

#### 5.2.3 Moderating effect analysis

As shown in Figure 3, technology acceptance significantly moderated the relationships between AI literacy and perceived autonomy (β=0.116,p=0.007), perceived competence (β=0.114,p=0.004), and perceived relatedness (β=0.199,p<0.001). These results provide empirical support for H11, H12, and H13. The findings suggest that higher levels of Technology Acceptance enhance the positive psychological impact of AI literacy. In other words, when teachers are more willing to embrace AI technologies, their AI literacy is more effectively translated into positive perceptions of autonomy, competence, and social connectedness.

**Figure 4:**
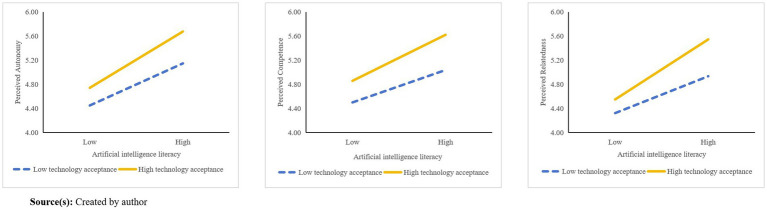
The moderating effect of technology acceptance.

To further examine and visually illustrate the moderating role of technology acceptance, this study plotted interaction diagrams (see Figure 4) depicting the relationships between AI literacy and perceived autonomy, competence, and relatedness at different levels of technology acceptance (low = −1 SD; high = +1 SD). As shown in Figure 4, a significant moderation effect was observed, wherein the positive pathways from AI literacy to basic psychological need satisfaction were amplified at higher levels of technology acceptance.

The results presented in Table 6 indicate that the effects of AI literacy on perceived autonomy (β=0.292,p<0.001), perceived competence (β=0.357,p<0.001), and perceived relatedness (β=0.226,p<0.001) are significant at low levels of technology acceptance. At moderate levels of technology acceptance, these effects become stronger for perceived autonomy (β=0.410,p<0.001), perceived competence (β=0.470,p<0.001), and perceived relatedness (β=0.418,p<0.001). At high levels of technology acceptance, the positive influence of AI literacy reaches its peak—perceived autonomy (β=0.528,p<0.001), perceived competence (β=0.583,p<0.001), and perceived relatedness (β=0.610,p<0.001). These findings suggest that the positive impact of AI literacy on individuals’ basic psychological needs becomes progressively stronger as technology acceptance increases, providing further support for hypotheses H11, H12, and H13.

**Table 6:** Moderating effect at different levels.

Independent variable	Dependent variable	TA	β	95%CI		*p*-value
AIL	PA	Low	0.292	0.176	0.407	***
		Medium	0.410	0.330	0.490	***
		High	0.528	0.421	0.635	***
	PC	Low	0.357	0.244	0.469	***
		Medium	0.470	0.392	0.547	***
		High	0.583	0.478	0.687	***
	PR	Low	0.226	0.118	0.333	***
		Medium	0.418	0.344	0.492	***
		High	0.610	0.510	0.710	***

*p < 0.05, **p < 0.01, ***p < 0.001.

Source(s): Created by author.

## 6 Discussion

Grounded in Self-Determination Theory (SDT), this study developed a structural model to investigate how AI literacy influences university faculty’s work–life balance and job satisfaction by satisfying three fundamental psychological needs: perceived autonomy, perceived competence, and perceived relatedness. Additionally, this study examined the moderating role of technology acceptance. The empirical results largely supported the proposed hypotheses, demonstrating that AI literacy is not merely a technical skill but a critical resource for activating intrinsic motivation and enhancing psychological well-being among teachers.

The findings revealed that AI literacy significantly and positively affects perceived autonomy, perceived competence, and perceived relatedness (H1–H3 supported), validating the pathway of “skill enhancement → psychological need satisfaction → motivational activation.” These results are consistent with prior studies (Ji et al., 2025; Pauw et al., 2022; Pelau et al., 2021), which emphasized that AI literacy not only improves technological performance but also strengthens teachers’ sense of instructional control, competence recognition, and social connectedness, ultimately enhancing their professional engagement.

Among the three psychological needs, perceived autonomy emerged as a significant positive predictor of both work–life balance and job satisfaction (H4 and H5 supported), corroborating Rahimi et al. (2024), who highlighted the pivotal role of autonomy in high-autonomy, high-demand professions. When teachers have greater decision-making power and scheduling flexibility in their use of AI technologies, they are better able to manage task pacing and balance multiple roles, thereby improving their job satisfaction (Deci et al., 2017).

In contrast, although perceived competence and perceived relatedness significantly enhanced work–life balance (H6 and H8 supported), their direct effects on job satisfaction were not significant (H7 and H9 not supported). One possible explanation lies in the evolving institutional and technological context of higher education. Many university performance evaluation systems still place greater emphasis on research output, grant acquisition, and autonomy in teaching innovation, while offering limited recognition for competence gains derived from technological adaptation (Singh et al., 2022). This focus may weaken the intrinsic satisfaction associated with improved competence, especially when such competence is perceived as an instrumental, externally driven requirement rather than a source of long-term professional pride (Lai and Jin, 2021; Wang et al., 2022). In AI-integrated teaching environments, competence increasingly reflects rapid adaptation and operational efficiency, characteristics that are transient and dependent on continuous technological updates, thereby reducing their potential to sustain job satisfaction (Zhou J. S. et al., 2024; Zhou T. et al., 2024). Similarly, the non-significant direct effect of perceived relatedness on job satisfaction may be linked to the functional limitations of current AI tools in fostering meaningful social connections. Although digital platforms facilitate communication, they tend to prioritize efficiency and task completion over relational depth (Yu, 2024; Zagni et al., 2025). As face-to-face collaboration is increasingly replaced by asynchronous or AI-mediated exchanges, opportunities for spontaneous peer support, emotional bonding, and informal knowledge sharing—critical components of professional fulfillment—are reduced (Turk et al., 2022). Furthermore, many AI tools in educational settings lack embedded collaborative features designed to build interpersonal trust and mutual support, thereby limiting their capacity to enhance relatedness in ways that translate directly into job satisfaction (Pelau et al., 2021). Taken together, these findings suggest that in technology-rich educational environments, competence and relatedness may contribute to job satisfaction mainly through indirect pathways—most notably by improving work–life balance—rather than through direct influence. This underscores the need for institutional reward systems that explicitly acknowledge competence development in technology adoption and for AI tools that integrate richer collaborative functions to strengthen professional relatedness (Rahimi et al., 2024; Singh et al., 2022).

Importantly, work–life balance significantly predicted job satisfaction (H10 supported), aligning with the findings of Landolfi et al. (2021), who emphasized the crucial role of life balance in constructing professional well-being. University faculty members who can effectively integrate their work and life roles are more likely to experience emotional stability and greater happiness. This result aligns with the work–family conflict framework outlined by Greenhaus and Beutell (1985), which posits that effective life balance can mitigate job stress and enhance satisfaction. Although perceived competence and perceived relatedness did not directly predict job satisfaction, they still exerted indirect effects through their positive contributions to work–life balance.

Furthermore, technology acceptance significantly moderated the relationships between AI literacy and the three basic psychological needs (H11–H13 supported). This finding is consistent with the “cognition–attitude–behavior” sequence in the technology acceptance Model (Belletier et al., 2018), suggesting that technology acceptance amplifies the positive psychological effects of AI literacy. Without sufficient confidence in and identification with AI tools, even teachers with high technical competence may struggle to fully activate psychological resources. Therefore, improving teachers’ AI literacy should be accompanied by efforts to enhance their technology acceptance, including providing scenario-based training, cognitive empowerment, and emotional support to strengthen their recognition of and willingness to engage with AI tools.

## 7 Contributions, limitations, and future research directions

### 7.1 Theoretical contributions

Grounded in Self-Determination Theory (SDT), the present research constructed a structural framework to examine how AI literacy shapes university faculty’s work–life balance and job satisfaction by fulfilling three fundamental psychological needs: perceived autonomy, perceived competence, and perceived relatedness. Additionally, the model integrated the technology acceptance as a moderating factor to systematically account for the psychological processes linking AI literacy to faculty well-being (Venkatesh et al., 2003). The theoretical contributions of this work are summarized in three key aspects:

First, this study extends the psychological conceptualization of AI literacy. Previous research has primarily regarded AI Literacy as an external technical competence or a performance indicator in educational settings (Celik, 2023; Chiu and Chai, 2020). In contrast, this study redefines AI Literacy as a psychological resource that activates intrinsic motivation. It demonstrates that AI Literacy can enhance work–life balance and job satisfaction by satisfying the psychological needs for autonomy, competence, and relatedness. The moderating effect of technology acceptance further reveals the critical role of cognitive attitudes in transforming technical literacy into motivational resources (Dahri et al., 2024), thereby expanding the theoretical boundaries and application pathways of AI literacy.

Second, this study reconstructs the pathway mechanisms and functional boundaries of SDT in high-technology environments. The findings indicate heterogeneous effects of the three psychological needs on faculty well-being: Perceived autonomy directly influences work–life balance and job satisfaction, while perceived competence and perceived relatedness primarily exert indirect effects through work–life balance (Ng et al., 2023; Van den Broeck et al., 2016). Furthermore, in AI-intensive teaching contexts, the direct impacts of competence and relatedness on job satisfaction were found to be non-significant, possibly due to the instrumental evaluation and function-oriented socialization patterns introduced by AI tools (Yu, 2024; Zagni et al., 2025). These results not only validate the context-dependency of SDT but also provide new insights for adapting and extending the theory in digital educational environments.

Third, this study advances the theoretical integration between educational technology and motivational psychology. The proposed integrated model—AI literacy → technology acceptance → psychological needs → job satisfaction—bridges the cognition–attitude mechanism of the technology acceptance Model with the motivation–well-being mechanism of SDT (Deci and Ryan, 2000; Venkatesh et al., 2003), offering a comprehensive framework to explain the interaction between individual behavior and psychological states in technology-driven settings.

### 7.2 Practical implications

This study offers the following practical recommendations for developing university faculty competencies and promoting the effective integration of educational technologies:

First, enhancing AI literacy should address both technical capabilities and psychological adaptability. Teacher training programs should not only develop operational skills and task optimization abilities but also cultivate the capacity for flexible technology transfer across varied instructional contexts, while fostering motivation and adaptability to change. Problem-based, project-oriented instructional activities can provide authentic problem-solving experiences, enabling teachers to build confidence and develop a sustained willingness to adopt AI tools.

Second, interventions should specifically target the indirect effects of competence and relatedness. Universities can integrate AI training with clear career development pathways, enabling teachers to enhance their skills while aligning them with professional growth trajectories, thereby strengthening competence satisfaction. At the same time, the design of collaborative AI-enabled teaching tools can promote cross-disciplinary resource sharing and foster the development of virtual academic communities, encouraging experience exchange and emotional connections that enhance relatedness and organizational belonging.

Third, it is essential to strengthen teachers’ technology acceptance to facilitate the internalization of AI literacy as a motivational driver. Role modeling, case-based learning, and experiential training can reduce uncertainty and enhance acceptance. Establishing “AI Empowerment Facilitator” roles or teacher learning communities can leverage positive peer influence to encourage proactive use of AI in teaching and research.

Fourth, teacher support policies should evolve toward personalized and continuous interventions. Recognizing differences in psychological responses across academic ranks, disciplines, and age groups, universities should implement stratified and targeted support systems. For example, younger faculty may benefit from growth-oriented feedback and belongingness support, while senior faculty may require greater flexibility, recognition, and opportunities for legacy building. Such tailored approaches can better align AI literacy development with career advancement goals.

### 7.3 Research limitations

The primary data source for this study was self-reported questionnaires completed by university faculty in Mainland China, which may introduce potential biases such as self-report bias and socially desirable responding. Although common method bias was tested using Harman’s single-factor method and found to be non-significant, the cross-sectional design limits the ability to capture dynamic changes and infer causal relationships over time. In addition, the current model did not control for or differentiate key job-related and demographic variables, such as teaching workload, academic rank, and disciplinary background, which may confound the relationship between AI literacy and job satisfaction. The absence of such controls limits the precision of the path estimates. Moreover, other unmeasured contextual variables (e.g., organizational support, institutional fairness, leadership styles, technology infrastructure) and personal characteristics (e.g., prior AI experience) may influence psychological need satisfaction and work-related outcomes, potentially affecting the model’s external validity.

### 7.4 Directions for future research

Future research should address these limitations in several ways. First, incorporating multi-source data—such as teaching logs, platform usage records, AI interaction trajectories, and classroom observations—would improve measurement validity and reduce the reliance on self-reported data. Second, adopting longitudinal or intervention designs would allow researchers to track the dynamic evolution of AI literacy and assess its long-term effects on teachers’ psychological states and professional well-being, thereby strengthening causal inference. Third, integrating multi-level contextual factors (e.g., organizational support, institutional fairness, leadership styles, technology infrastructure) and individual difference variables (e.g., academic rank, disciplinary background, prior AI experience) as covariates in the structural model would refine the precision of the estimates and illuminate potential moderating mechanisms. Finally, subgroup analyses could identify heterogeneous pathways in psychological need satisfaction across different teacher groups, offering tailored evidence for faculty development policies and technology-driven interventions.

## Data Availability

The raw data supporting the conclusions of this article will be made available by the authors, without undue reservation.

## Footnotes

^1^ https://www.wjx.cn/vm/eAGu0vk.aspx
