# Supplementary material for: The impact of AI literacy on work–life balance and job satisfaction among university faculty: a self-determination theory perspective
Source: Front Psychol. 2025 Sep 17;16:1669247. doi: 10.3389/fpsyg.2025.1669247 (PMC12487956; doi:10.3389/fpsyg.2025.1669247)
Supplement: Supplementary file 1 [file Table_1.DOCX]

**Appendix 1. Measurement Scales**

| Variable | Item | Item Descriptio | Scale Source |
| --- | --- | --- | --- |
| AI Literacy (AIL) | AIL1 | I can distinguish AI devices from non-AI devices. | (Wang et al., 2023) |
|  | AIL2 | I can identify the AI technologies used in the applications or products I encounter daily. |  |
|  | AIL3 | I understand and can use AI tools to enhance my teaching or research efficiency. |  |
|  | AIL4 | I am proficient in using AI-related applications or products to accomplish teaching or research tasks. |  |
|  | AIL5 | I can select the most suitable AI tool or platform based on specific task requirements. |  |
|  | AIL6 | I can assess both the strengths and limitations of AI applications. |  |
|  | AIL7 | When an intelligent system offers several solutions, I can choose the most appropriate one. |  |
|  | AIL8 | I actively consider ethical and privacy issues when using AI tools. |  |
|  | AIL9 | I remain alert to potential misuse of AI technologies in teaching or research. |  |
| Perceived Autonomy (PA) | PA1 | My organization encourages me to express my opinions in important career decisions. | (Haw et al., 2024) |
|  | PA2 | When I disagree with a decision, management encourages me to voice my views. |  |
|  | PA3 | My suggestions and feelings are taken seriously at work. |  |
|  | PA4 | Supervisors consider employees’ diverse viewpoints before making decisions. |  |
|  | PA5 | I am free to choose teaching or research methods that suit me. |  |
| Perceived Competence (PC) | PC1 | My organization is concerned about whether I achieve my career goals. | (Haw et al., 2024) |
|  | PC2 | I receive proper recognition and feedback for important accomplishments. |  |
|  | PC3 | I obtain constructive advice that helps improve my teaching or research ability. |  |
|  | PC4 | When I need to enhance my skills, the organization provides training or learning opportunities. |  |
|  | PC5 | I am confident in my ability to handle my current teaching and research duties. |  |
| Perceived Relatedness (PR) | PR1 | Colleagues and managers treat every teacher fairly. | (Haw et al., 2024) |
|  | PR2 | My interests are respected and protected by the organization. |  |
|  | PR3 | My supervisor is someone I can trust. |  |
|  | PR4 | I build trusting and positive relationships with colleagues. |  |
|  | PR5 | I feel accepted and valued within the organization. |  |
| Work–Life Balance (WLB) | WLB1 | I can flexibly schedule my work time to accommodate personal or family needs. | (Mulyani et al., 2021) |
|  | WLB2 | My current work schedule matches my life rhythm and personal needs. |  |
|  | WLB3 | When necessary, I can easily adjust my work schedule or take leave for personal matters. |  |
|  | WLB4 | My organization supports employees in attending to personal or family matters when needed. |  |
|  | WLB5 | My unit encourages employees to maintain a balance between work and life. |  |
|  | WLB6 | My organization respects employees’ personal and family life needs. |  |
|  | WLB7 | My supervisor shows understanding and support when I face personal or family difficulties. |  |
|  | WLB8 | My supervisor is willing to accommodate schedule adjustments I need for family matters. |  |
| Job Satisfaction (JS) | JS1 | I am satisfied with my job overall. | (Yildirim et al., 2024) |
|  | JS2 | My work makes me feel valuable and accomplished. |  |
|  | JS3 | I go to work each day with enthusiasm. |  |
|  | JS4 | I find my work highly interesting. |  |
|  | JS5 | My job allows me to fully utilize my abilities. |  |
| Technology Acceptance (TA) | TA1 | I believe AI teaching tools help enhance my teaching effectiveness. | (Lin et al., 2025) |
|  | TA2 | Using AI teaching tools increases my teaching or research efficiency. |  |
|  | TA3 | AI tools strengthen my ability to deliver differentiated instruction. |  |
|  | TA4 | I find AI teaching tools easy to operate and understand. |  |
|  | TA5 | Learning to use AI teaching tools is an effortless process for me. |  |
|  | TA6 | AI teaching tools can be easily integrated into my existing teaching workflows. |  |
|  | TA7 | I am concerned that AI tools may introduce algorithmic bias during use. |  |
|  | TA8 | I think excessive reliance on AI tools could weaken teachers’ professional judgment. |  |
|  | TA9 | I am concerned that AI systems may produce errors in data processing or calculations. |  |
| **Source(s):** Created by author | | | |
